# Harnessing on Genetic Variability and Diversity of Rice (*Oryza sativa* L.) Genotypes Based on Quantitative and Qualitative Traits for Desirable Crossing Materials

**DOI:** 10.3390/genes14010010

**Published:** 2022-12-21

**Authors:** Sanjoy K. Debsharma, Md. Abu Syed, Md. Hannan Ali, Sheikh Maniruzzaman, Popy R. Roy, Marian Brestic, Ahmed Gaber, Akbar Hossain

**Affiliations:** 1 Plant Breeding Division, Bangladesh Rice Research Institute, Gazipur 1701, Bangladesh; 2 Irrigation and Water Management Division, Bangladesh Rice Research Institute, Gazipur 1701, Bangladesh; 3 Seed Certification Agency, Ministry of Agriculture, Government of the People’s Republic of Bangladesh, Gazipur 1701, Bangladesh; 4 Institute of Plant and Environmental Sciences, Slovak University of Agriculture, Tr. A. Hlinku 2, 949 01 Nitra, Slovakia; 5 Department of Biology, College of Science, Taif University, P.O. Box 11099, Taif 21944, Saudi Arabia; 6 Division of Soil Science, Bangladesh Wheat and Maize Research Institute, Dinajpur 5200, Bangladesh

**Keywords:** genetic diversity, trait association, principal component analysis, clustered heatmap, MGIDI

## Abstract

Yield is a complex parameter of rice due to its polygonal nature, sometimes making it difficult to coat the selection process in the breeding program. In the current study, 34 elite rice genotypes were assessed to evaluate 3 locations for the selection of desirable rice cultivars suitable for multiple environments based on genetic diversity. In variance analysis, all genotypes have revealed significant variations (*p* ≤ 0.001) for all studied characters, signifying a broader sense of genetic variability for selection purposes. The higher phenotypic coefficient of variation (PCV) and genotypic coefficient of variation (GCV) were found for yield-associated characteristics such as the number of grains panicle^−1^ (GP), panicles hill^−1^ (PPH), and tillers hill^−1^ (TILL). All of the characters had higher heritability (greater than 60%) and higher genetic advance (greater than 20%), which pointed out non-additive gene action and suggested that selection would be effective. The most significant traits causing the genotype variants were identified via principal component analysis. In the findings of the cluster analysis, 34 elite lines were separated into 3 categories of clusters, with cluster II being chosen as the best one. The relationship matrix between each elite cultivar and traits was also determined utilizing a heatmap. Based on multi-trait genotype-ideotype distance index (MGIDI), genotypes Gen2, Gen4, Gen14, Gen22, and Gen30 in Satkhira; Gen2, Gen6, Gen7, Gen15, and Gen30 in Kushtia; and Gen10, Gen12, Gen26, Gen30, and Gen34 in Barishal were found to be the most promising genotypes. Upon validation, these genotypes can be suggested for commercial release or used as potential breeding material in crossing programs for the development of cultivars suitable for multiple environments under the future changing climate.

## 1. Introduction

Rice (*Oryza sativa* L.) is a unique, particularly crucial cereal; over half of the world’s population relies on rice. In Bangladesh, around 158.9 million individuals rely mostly on rice as their main food source [1]. The absolute rice region in Bangladesh is 11.52 million ha and accounts for 75% of the entire crop area and 93% of the entire area cultivated for cereals. The overall rice production in Bangladesh was 36.4 MMT (Million Metric Tons), of which Boro, Aman, and Aus rice occupied 19.5 MMT (53.8%), 14.1 MMT (38.6%), and 2.8 MMT (7.6%) respectively, during 2018–2019 [2]. In contrast with many other rice-producing countries, Bangladesh also has ‘rice security’ as being synonymous with ‘food security’ [3]. The safety of rice production is not only a socio-economic problem but also an essential indicator of the stability of political and cultural harmony [4]. It is estimated that the individuals of Bangladesh will reach 215.4 million by the year 2050, and at the same time, 44.6 MMT of rough rice would be needed to secure the food sufficiency of the rising population [5]. Therefore, the main objectives of plant breeders should be to develop climate-smart varieties as well as yield improvement for the main food crop. Hence, the improvement of high-yielding rice genotypes is expected to be met with agronomic characteristics and the need to cope with the growing demand associated with adaptability in different environments. However, the complex polygenetic trait of the yield, which is influenced by its inherent properties, confuses plant breeders for its direct selection [6,7]. It is crucial to understand how yield relates to its other inherent characteristics [8]. The vast spectrum assortment of agricultural climates, physiological, soil, and hydrological circumstances makes this country a great source of local land racing, and especially, an excessive source of rice with distinguishing phenotypic and genetic characteristics [9]. Discovering this existing diversity in the rice germplasm certainly leads to identifying the fancy genes and boosting the progress in rice breeding [10]. Before selecting the applied breeding method in rice, it is indispensable to know the morpho-genetic dissimilarity in diverse yield-related characters [11,12]. Although there are high environmental effects, assessments based on morphological characteristics are still possible for selection [13,14]. Attributes such as the GCV, PCV, heritability, and genetic advance are convenient biometric aspects for evaluating genetic variability and adaptability [15,16]. By utilizing more genetic varieties and employing efficient selection techniques to boost yield through yield attributes, breeding projects seek to increase rice production. The correlation studies help plant breeders make more accurate selections. Due to the rising importance of crop development, current knowledge of trait connections aids in the proper selection process [17].

Diversity analysis is a tool that helps to divide germplasms into several clusters based on their similar performance in various characteristics [18,19]. Previous literature suggests that there is more genetic dissimilarity in rice for yield traits and its associated characteristics in Bangladesh [20,21]. Nevertheless, even thousands of significant novel genes with economic value remain undiscovered. Therefore, these dissimilarities need to be explored to face current and future challenges in rice breeding. The MGIDI [22] has become one of the most innovative techniques for choosing the best genotypes that might perform much better under various favourable and unfavourable conditions with high yield stability and desired characteristics. Thus, the current investigation was carried out to uncover the genetic divergence and genetic diversity of 34 elite rice genotypes across qualities and agronomic traits for future rice breeding programs for the development of superior and stable cultivars which are suitable for multiple environments under the future changing climate.

## 2. Materials and Methods

### 2.1. Experimental Locations and Weather Data

To assess the genotypes, the research was conducted in three representative environments of Bangladesh, namely Barishal (22°53′ N and 90°34′ E), Kushtia (23°55′ N and 89°03′ E), and Sathkira (22°76′ N and 89°11′ E). The monthly mean temperatures, wind speed, and precipitation data during the experimental period from February to May 2017 were presented in Table 1.

### 2.2. Plant Genetic Materials

Thirty-four genotypes containing twenty-six elite breeding lines and eight BRRI-released varieties such as BR16 (released in 1983), BRRI dhan28 (1994), BRRI dhan29 (1994), BRRI dhan50 (2008), BRRI dhan58 (2012), BRRI dhan63 (2014), BRRI dhan74 (2015), and one International Rice Research Institute (IRRI) developed variety; IRBB60 were used. All genetic materials were collected from the Plant Breeding Division, Bangladesh Rice Research Institute (www.brri.gov.bd; accessed on 22 July 2022). The details of those genotypes are illustrated in Table 2.

### 2.3. Design of Experiment and Agronomic Practices

The plant material utilized in the current investigation is made up of 34 genotypes of rice accessed from the BRRI (www.brri.gov.bd; accessed on 22 July 2022). All the tested breeding lines were grown in dry seasons in the three experimental fields at BRRI Barishal, BRRI Khustia, and BRRI Satkhira, Bangladesh. The study was evaluated by utilizing the randomized complete block design with three blocks in each experimental site. Thirty-five to forty-day-old seedlings were placed in the experimental field in a 10.8 m^2^ plot utilizing one seedling at 20 cm × 15 cm spacing. The fertilizers were supplied at 260:100:120:110:10 kg ha^−1^ of urea, TSP, MP, gypsum, and ZnSO_4_, respectively. Standard cultural management was practiced and plant protection measures were taken as required according to the suggestion of BRRI [23]. The extra two lines were kept in the plot to lessen the effects of plant growth as well as yield production.

### 2.4. Data Collection of Studied Agronomic Traits

A standard evaluation system (SES, 2013) developed from IRRI was followed to collect data on agronomic characters [24]. Data were gathered on 50% flowering (X50% F, days), plant height (PH, cm), tillers per plant (TILL, no.), number of panicles per hill (PPH), panicle length (PL, cm), grains per panicle (GP, no.), fertility (Ferti, %), grain yield per plant (YPP), 1000-grain weight (TGW, gm), and yield ton per hectare (YTH, t/ha). Days to flowering have been collected as early as when 50% of the panicles appear. The five plants were haphazardly counted, eliminating border plants and plants adjacent to the missing hill, for plant height in terms of centimetre units. The measurement of a plant’s height was taken from the base to the top of the panicle. The number of tillers was collected when the grain had been set and the total number of fertile panicles emerged from each plant. The panicles were harvested after maturity and placed in an envelope separately. The harvested panicles were dried on a threshing floor utilizing sunlight. After that, the number of filled and unfilled grains was counted, and thousand grain-weight and grain yield per plant were measured from harvested panicles. Each unit plot was harvested and then dried on the floor by sunlight. Finally, the plot grain yield was weighed and calculated as a ton per hectare, where moisture was adjusted at 14%.

### 2.5. Statistical Analysis

#### 2.5.1. Estimations of Variance Components

The variation that exists among the germplasm was measured by measuring mean, phenotypic, and genotypic variance. The mean values of each genotype were utilized for combined analysis of variance following STAR software (Version 2.0.1; http://bbi.irri.org/products; accessed on 20 July 2022).

To evaluate the phenotypic and genotypic variance, GCV and PCV were assessed and marked to utilize the formula suggested by Johnson et al. [25]. Broad sense heritability (h^2^bs) of all desired characters was determined using the equation as depicted by Allard [26]. Heritability was categorized with a value of 0–10% as low, 10–20% as moderate, and 20% to above as high, which was reported by Burton and DeVane [27]. Genetic Advance (GA) was calculated as interpreted in the equation by Singh and Chaundry [28]:

#### 2.5.2. Correlation Coefficients Analysis

The correlation matrix was created from chart.Correlation()with the “PerformanceAnalytics” package in R.

#### 2.5.3. Multivariate Analysis

PCA was executed to calculate eigenvalues, association between cultivars and/or traits, and contributions with quality of traits and genotypes towards principal components of the total variation. The multivariate analysis was performed utilizing R (v4.1.1) and R Studio software (v1.4.1717). The PCA–biplot was assembled using “ggplot2”, “Factoextra”, and “FactomineR” packages of R. A package, namely NbClust, which was utilized to measure the optimal cluster number in a dataset. NbClust contains 30 different valid indices, namely “KI”, “CH”, “Hartigan”, “CCC”, “Scott”, “Marriot”, “TrCovW”, “TraceW”, “Friedman”, “Rubin”, Cindex”, “DB”, “Silhouette”, “Duda”, Pseudot2”, “Beale”, “Ratkowsky”, “Ball”, “Ptibiserial”, “Gap”, “Frey”, “McClain”, “γ”, “Gplus”, “Tau”, “Dunn”, “Hubert”, “SDindex”, “Dindex”, and “SDbw”. The cluster plot with heatmap was visualized with the “heatmaply” package of R.

#### 2.5.4. Analysis of MGIDI Selection Index

The statistical analysis for the multi-trait genotype–ideotype distance index (MGIDI) was performed utilizing R Package ‘metan’ version 1.16.0 (https://github.com/TiagoOlivoto/metan; accessed on 20 July 2022) in R version 4.0.2 (http://www.r-project.org/; accessed on 20 July 2022) [29].

## 3. Results

### 3.1. Genetic Variability Analysis

The variability analysis for different locations, as well as pooled data, exhibited significant (*p* ≤ 0.01) variation along the rice genotypes for all the traits evaluated (Table 3). The interaction variance between genotypes × locations was found to be significant for the traits, illustrating the consistent performance of the genotypes across the year with a significant effect of environmental factors.

The estimations of range, mean value, variance components, heritability, and genetic advance for various traits with other statistical indicators are shown in Table 4. The days of 50% flowering varied from 103 to 135 with an average of 118.2 ± 0.9 in Barishal, 110 to 129 with an average of 121 ± 0.8 in Kushtia, while in Satkhira, it varied from 106 to 124 days with an average of 114.7 ± 0.8. The range of plant height varied from 80 to 148 cm with an average of 105.7 ± 3.4 in Barishal, 77 to 131 cm with an average of 99 ± 2.6 in Kushtia, while it ranged from 81 to 124 cm with an average of 101.2 ± 2.5 in Satkhira (Table 4).

The range of TILL varied from 8 to 23 with a mean of 13.8 ± 1.5 in Barishal; in Kushtia, it varied from 5 to 18 with an average of 15.7 ± 2.2; and in Satkhira, it ranged from 7 to 18 with a mean 11.2 ± 0.7. The PP varied from 8 to 23 (average 12.5 ± 1.4), 5 to 18 (average 10.4 ± 1.5), and from 6.3 to 16.5 (average 10.7 ± 0.9) in Barishal, Kushtia, and Satkhira, respectively. The panicle length varied from 20 to 27.9 cm with a mean value of 23.9 ± 0.7 in Barishal, 19 to 30 cm with an average value of 24.4 ± 1.3 in Kushtia, and 18.5 to 29.8 cm with a mean value of 23.8 ± 0.7 at the Satkhira location. The grains per panicle were found to be variable between 151 and 607 (average 307.3 ± 49.6) in Barishal, between 160 and 484 (average 267.5 ± 23.5) in Kushtia, and between 79 and 375 (average 210 ± 25.5) in Satkhira. The yield per plant was found to range from 16.9 to 32.5 gm with an average of 24.2 ± 1.2 in Barishal, and a range from 18 to 37.5 gm with an average of 28.6 ± 1.9 in Kushtia, while the range was from 17.6 to 28.9 gm with an average of 22.7 ± 1.1 in Satkhira. The range of 1000-grain weight varied from 15.7 to 33 gm with an average of 21.8 ± 1.2 in Barishal, 16.9 to 31.2 gm with an average of 22.1 ± 0.7 in Kushtia, while it ranged from 13.8 to 28.9 gm with an average of 20.7 ± 1.5 in Satkhira. The yield tons per hectare varied from 4.5 to 8.6 (average 6.41 ± 0.4), 4.4 to 9.3 (average 7.04 ± 0.5), and from 4.6 to 7.9 (average 6.24 ± 0.5) in Barishal, Kushtia, and Satkhira, respectively. It was noticed that the average value of yield (t/ha) for Kushtia was higher than in the other two locations.

Various genetic factors were evaluated to determine the genetic variability for specific features that already existed among the genotypes (Table 4). The phenotypic variance was divided into genotypic and error variance to evaluate the heritable component of overall variability. These results clearly showed that variability in the genotypes was primarily because of the genotypic variance, as the error variance units were low in three locations. The PCV values for all the characters were found to be higher than the corresponding GCV. The GP, TILL, and PPH recorded high values of GCV and PCV, while days to X50F, PL, and PH showed the lowest values in three locations (Table 4). Broad sense heritability was high for the days to X50F (99.4%), followed by PH (95.6%), TGW (94.8%), and fertility percentage (93.5%) in Barishal. At the Kushtia and Satkhira locations, these traits also showed higher heritability. The traits PP (62.5%), PL (72.8%), and TILL (76.7%) showed the lowest heritability in Kushtia. In Barishal and Satkhira, the genetic advance was found to be highest for GP at (55.6%) and (43.3%), followed by PP at (58.4%) and (34%), respectively. The highest number of GP (39.8%) was followed by the TILL (32.5%) in Kushtia. The days to 50F and PL had the lowest GA in the three locations.

### 3.2. Correlation Matrix among the Traits over Locations

**Figure 1:**
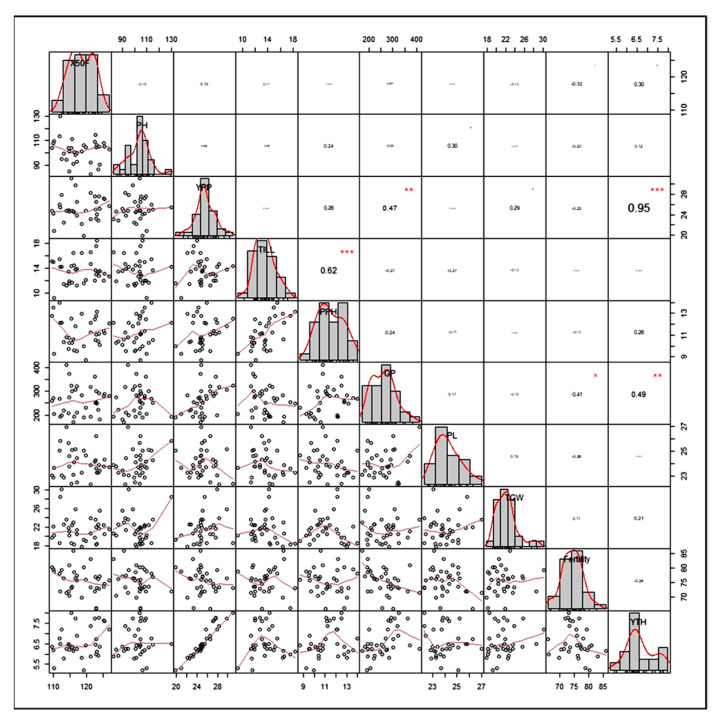
Correlation coefficient matrix, scatter plot, and phenotypic frequency distribution among grain yield and yield-related traits over the locations. Each variable’s distribution is displayed diagonally. The bivariate scatter plots with a trend line are shown at the bottom of the diagonal. The correlation coefficient and the level of significance are displayed as stars at the top of the diagonal. * *p* ≤ 0.05, ** *p* ≤ 0.01, and *** *p* > 0.001 show significance level; Note: X50F = days to 50% flowering, PH = plant height (cm), TILL = number of tillers per hill, PPH = number of panicles per hill, PL = panicle length (cm), GP = grains per panicle, Fertility = fertility (%), YPP = grain yield per plant (gm), TGW = thousand grain weight (gm), and YTH = grain yield (t/ha).

To comprehend the degree of relationship among the investigated variables and to highlight their significance in rice breeding programs, Pearson’s correlation coefficient matrix was displayed in Figure 1.

The findings exhibited that YTH showed a positive and highly significant correlation along with YPP (0.95 ***) and GP (0.49 **). Fertility (%) was positively associated with TILL (0.02) and TGW (0.11), but there was a negative and significant association with GP (−0.41 *). The TGW had a positive association with YPP (0.29) and PL (0.15). The PL was positively correlated with PH (0.30) and GP (0.17). The GP showed a highly significant positive association with YPP (0.47 **), but a negative correlation with the TILL (−0.27). The PPH revealed a highly significant positive correlation with the TILL (0.62 ***), but only a positive correlation with YPP (0.26) and PH (0.24).

### 3.3. Analysis of Principal Components of the Studied Traits

#### 3.3.1. Graphical Presentation of the Scree Plot

**Figure 2:**
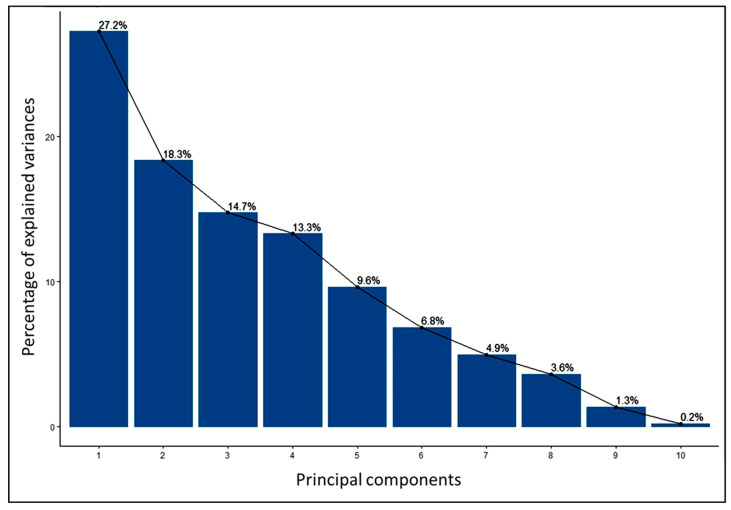
Scree plot on variability explained by each component of 34 elite rice genotypes.

The Scree plot described the percentage of variation for the respective principal components (Figure 2). PC1 to PC10 explained 100% of the variance. PC1 showed the maximum amount (27.2%) of the total variance, whereas PC2 depicted 18.3%. The other PC3 to PC10 exhibited 14.7, 13.3, 9.6, 6.8, 3.6, 1.3, and 0.2% of the total variance, respectively. The first five PCs contributed above 80% of the total variability. As a result, five PCs can be used to demonstrate the contribution and quality of top-performer genotypes and variables to total variability.

#### 3.3.2. PCA-Biplot Analysis

**Figure 3:**
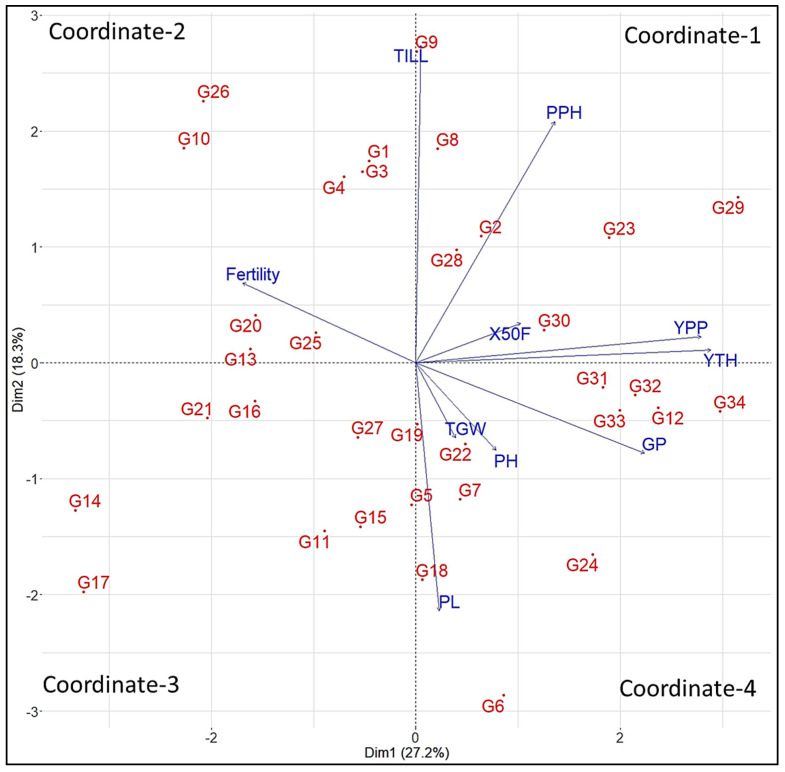
Biplot for the 34 rice genotypes and 10 agronomic traits along the first 2 principal components. Note: X50F = days to 50% flowering, PH = plant height (cm), TILL = number of tillers per hill, PPH = number of panicles per hill, PL = panicle length (cm), GP = grains per panicle, Fertility = fertility (%), YPP = grain yield per plant (gm), TGW = thousand grain weight (gm), and YTH = grain yield (t/ha).

The biplot analysis shows the relationship among the various traits and genotypes concerning the first two principal components, which exhibited 45.5% of divergence in the dataset (Figure 3). In the biplot, the two first principal components were visualized into four coordinates along the *x-* and *y*-axis. Coordinate-1 is comprised of seven genotypes, namely, Gen2, Gen8, Gen9, Gen23, Gen28, Gen29, and G30, with the positive value of the first and second PCs. This coordinate’s genotypes were associated with important variables such as the TILL, PPH, X50F, YPP, and YTH. Gen29 is located far from the origin and has the greatest diversity, followed by Gen23, whereas other genotypes are located near the centre. The grain yield (t/ha) exhibited the longest vector, followed by grain yield per plant, which reflected the greater variance.

The genotypes such as Gen1, Gen3, Gen4, Gen10, Gen13, Gen20, Gen25, and Gen26 are plotted in the direction of negative values of PCA1 and positive values of PCA2 in coordinate-2, where the percent of the fertility trait is strongly associated with those genotypes. The first two principal components were negative in the eight genotypes placed at coordinate 3. The maximum number of genotypes (11) is located in coordinate-4, which had positive and negative values of the first and second PCs, respectively. The characteristics such as PL, GP, TGW, and PH are largely contributed to by the 11 genotypes. Among the four variables, GP showed the longer line that reflected the greater variance. The distribution of genotypes in the biplot represented that there was some genetic variability among the different rice breeding lines.

#### 3.3.3. Contribution along with the Quality of Variables and Genotypes

**Figure 4:**
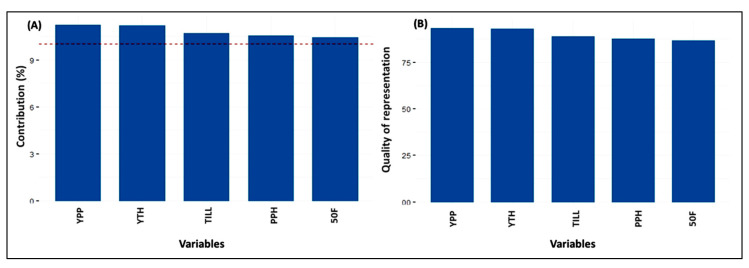
Graphical representation on (**A**) percent of contribution and (**B**) quality of representation of top 5 variables to principal components 1-2-3-4-5.

**Figure 5:**
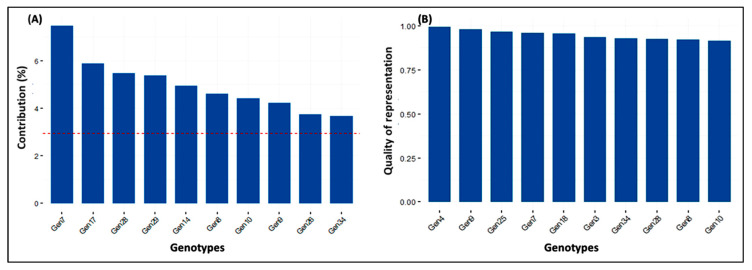
Graphical representation of (**A**) percent of contribution and (**B**) quality of representation of top 10 genotypes to principal components 1-2-3-4-5.

Among the ten variables, the maximum % of contribution and quality of representation towards the total variability was exhibited in the YPP, succeeded by YTH, TILL, PPH, and X50F to principal components 1-2-3-4-5 (Figure 4). In the 34 genotypes, the highest percentage of contribution towards the entire diversity was revealed in the genotype Gen7, followed by genotypes Gen17, Gen28, Gen29, Gen14, Gen8, Gen10, Gen9, Gen26, and Gen34, respectively, to principal components 1-2-3-4-5. However, the genotypes of Gen4 showed the maximum quality divergence, followed by Gen9, Gen25, Gen7, Gen18, Gen3, Gen34, Gen28, Gen8, and Gen10, respectively (Figure 5).

### 3.4. Analysis of Clusters among Traits

#### 3.4.1. Choice of the Best Cluster Number

A total of 30 indices were utilized to identify the best cluster number during NbClust analysis. A total of 28 indices represented the number of clusters based on index value (Table 5).

**Figure 6:**
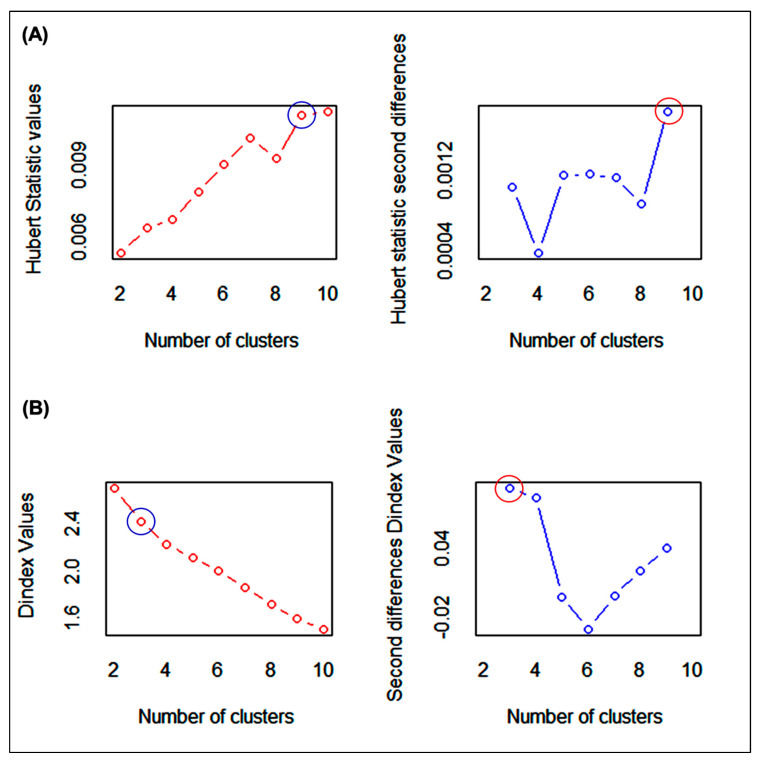
Graphical method (**A**) Hubert index and (**B**) Dindex for detecting the optimum number of clusters.

The other two methods, Huber and Dindex, are graphical methods that consider a significant knee for index value and a significant peak for second differences plotted. According to significant knee and peak value, nine is the optimal number of clusters in the Hubert index and three is the best number of clusters in the Dindex method (Figure 6). The results showed that five indices suggested two as the best number of clusters, eight indices recommended three as the best number of clusters, one index recommended four as the best number of clusters, two indices suggested five as the best number of clusters, one index recommended six as the best number of clusters, one index recommended seven as the best number of clusters, four indices suggested eight as the best number of clusters, one index recommended nine as the best number of clusters, and seven indices recommended ten as the best number of clusters. The best number of clusters, as determined by the majority rule, is three (Figure 6). Therefore, the initial value of k = 3 would be utilized to execute the K-means cluster analysis.

#### 3.4.2. Grouping of Genotypes by Heatmap Oriented Clustering Pattern

**Figure 7:**
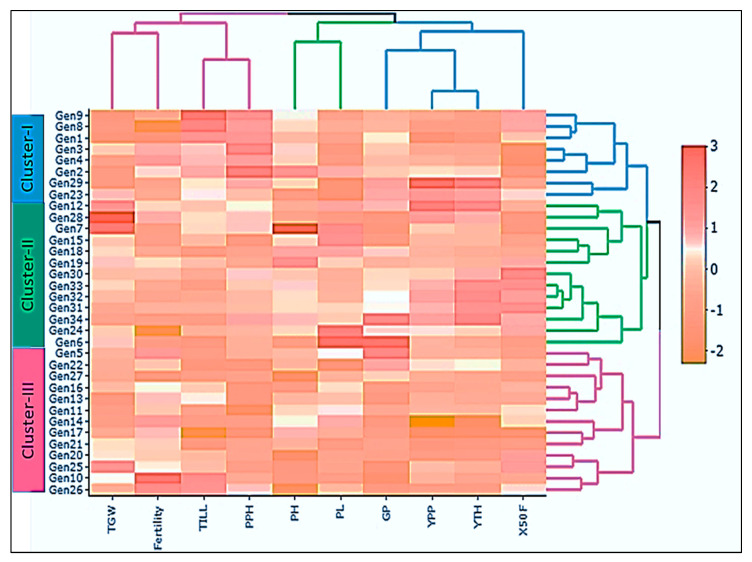
The heatmap displays the clustering pattern of 34 rice genotypes with 10 quantitative characters. Each column indicates a character, whereas each row represents a genotype. The various colours and intensities (−2 to 3) were adjusted based on the genotype–characters relationship. The orange colour represents a lower value, the white colour for mid value, and the dark red indicates a higher value.

A heatmap is a two-dimensional data visualization approach that employs the use of colour to depict a phenomenon’s extent. The reader can see how the phenomenon is grouped or changes over space by looking at colour variation by intensity. It shows the relative distribution of highly expressed characteristics over a background of predominantly small features (Figure 7). As a result, the heatmap analysis generated two dendrograms: one in the horizontal direction reflecting the features that triggered the diffusion and one in the vertical direction representing the germplasm accessions.

In the present study, heatmap-oriented cluster analysis using average values of all the studied traits of 34 rice genotypes was conducted, and genotypes were classified into 3 clusters. The frequency pattern of clusters exhibited that a large number of genotypes (13) was found in cluster II and cluster III, whereas the lowest number of genotypes (8) was situated in cluster I (Table 6 and Figure 7).

Cluster I genotypes are BR7671-37-2-2-37-3-P3 (Gen1), BR8626-19-5-1-2 (Gen2), BR8626-10-5-1(Gen3), BR8109-29-2-2-3 (Gen4), BR(Bio)8333-BC5-2-22 (Gen8), BR(Bio)8333-BC5-3-10 (Gen9), BRC266-5-1-2-1 (Gen23), and BRRI dhan58 (Gen29). Cluster II contains 13 genotypes: BR(Bio)8333-BC5-1-20 (Gen6), BR(Bio)8333-BC5-2-16 (Gen7), BR7831-59-1-1-4-5 (Gen12), BR7815-18-1-3-2-1 (Gen15), BR8079-19-1-5-1 (Gen18), BR8590-5-2-5-2-2 (Gen19), BR8523-36-2-2-6 (Gen24), BRRI dhan28 (Gen28), BRRI dhan29 (Gen30), BR16 (Gen31), BRRI dhan50 (Gen32), BRRI dhan63 (Gen33), and BRRI dhan74 (Gen34). Cluster III contains 13 genotypes, including BR (Bio) 8333-BC5-1-1 (Gen5), BR8631-12-3-5-P2 (Gen10), BR8631-12-3-6-P3 (Gen11), BR8253-9-3-3-1 (Gen13), BR8609-2-B-9-1-B5 (Gen14), BR7671-37-2-2-3-7-3-P10 (Gen16), BR7671-37-2-2-3-7-3-P11 (Gen17), BR8590-5-3-3-4-2 (Gen20), BR8608-39-2-1 (Gen21), BRC266-5-1-1-1 (Gen22), BR8938-19-4-3-1-1 (Gen25), BR8333-15-3-2-2 (Gen26), and IRBB60 (Gen27). Other dendrograms revealed three distinct groups. Group-1 is linked with four characters (TGW, Fertility, TILL, and PPH). Group-2 is connected with two characters (PH and PL). Group-3 is allied with four characters (GP, YPP, YTH, and X50F) (Figure 7).

### 3.5. Identification of the Best Performing Genotypes through Multi-Trait Genotype–Ideotype Distance Index (MGIDI)

**Figure 8:**
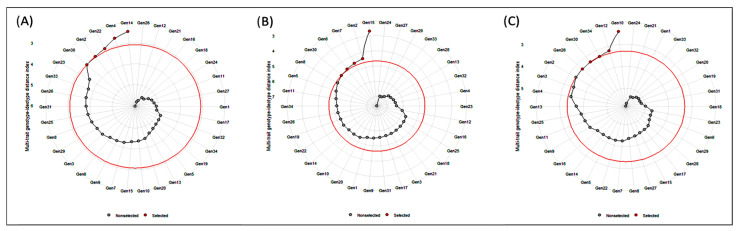
The circular preview indicates the ranking of genotypes based on the MGIDI selection index as well as the best rice genotypes with the associated locations (**A**) Satkhira, (**B**) Kushtia, and (**C**) Barishal. The selected ones are marked in red colour.

The multi-trait genotype–ideotype distance index (MGIDI) was used to select the best-performing genotypes utilizing the MGIDI selection index. The selection outputs of different locations are presented accordingly. A selection difference in percent (DS) of 16.3% for GP is highlighted followed by 12.4% for YPP in Satkhira (Table 7), 11.9% for YTH is compared to 11.4% for YPP in Kushtia (Table 8), and 12.6% for PPH is highlighted followed 8.31% for TILL in Barishal in the selection gain analysis results (Table 9) from MGIDI analysis, which revealed gains in the desired sense in 34 genotypes of the 10 characters. A total of 34 genotypes, 5 genotypes in each location, e.g., genotypes Gen2, Gen4, Gen14, Gen22, and Gen30 in Satkhira; Gen2, Gen6, Gen7, Gen15, and Gen30 in Kushtia; and Gen10, Gen12, Gen26, Gen30, and Gen34 in Barishal were selected. These genotype performances went well, which is equivalent to the ideotype utilized in MGIDI. However, because of their superior performance, genotypes Gen28 and Gen12 in Satkhira, Gen24 and G27 in Kushtia, and Gen24 and Gen21 in Barishal displayed greater sensitivity (Figure 8).

**Figure 9:**
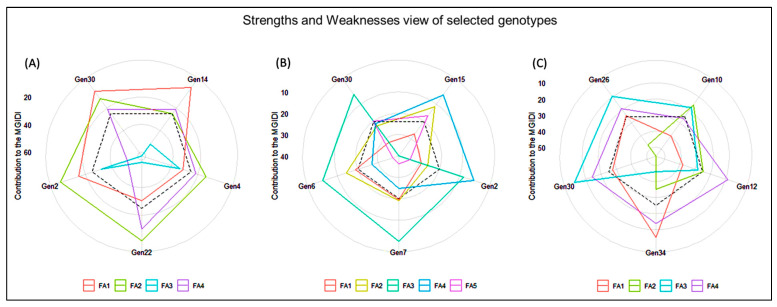
The strengths and weaknesses view of the selected genotypes represent the proportion of each factor on the computed MGIDI index. (**A**) Satkhira, (**B**) Kushtia, and (**C**) Barishal.

In the Satkhira region, regarding the strengths and weaknesses of the five selected genotypes (Figure 9A and Table 7), we found the Gen4 lineage with a strong contribution to factor 1 (FA1: PPH, GP and TGW) and a weak contribution of Gen14. For factor 2 (FA2: X50F, PH, and TILL), Gen14 lineage observed strong and weak contributions of Gen2. In factor 3 (FA3: YPP, and YTH), the Gen30 lineages were observed as strong and Gen2 presented a weak contribution, and in factor 4 (FA4: PL and Fertility), the Gen2 lineage revealed a strong contribution in this factor.

In Kushtia, for factor 1 (FA1: YPP and YTH), Gen30 were strong and Gen6 were a weak contributor (Figure 9B and Table 8). To factor 2 (FA2: TILL, PPH, and GP), the Gen30 lineage presented strong and weak participation of Gen15. For factor 3 (FA3: X50F and PH), the Gen15 lineage noticed strong and weak points of Gen7. For factor 4 (FA4: TGW), the genotypes Gen6 and Gen15 have strong and weak points, respectively. The Gen6 genotypes were strong and the Gen15 genotypes were weak contributions in factor 5 (FA5: PL and Fertility).

In Barishal, for factor 1 (FA1: YPP and YTH), Gen10 were strong and Gen34 was a weak contributor (Figure 9C and Table 9). For factor 2 (FA2: TILL and PPH), the Gen30 lineage presented strong and weak participation of Gen10. For factor 3 (FA3: PH, PL, Fertility and GP), the Gen12 lineage revealed strong and weak points of Gen30. In factor 4 (FA4: X50F and TGW), the genotypes Gen10 and Gen12 have strong and weak points.

## 4. Discussion

### 4.1. Variability Analysis among the Studied Traits

Genetic variations in crop gene-pool resources can be displayed using morphological markers easily and straightforwardly [30]. The assessment of genetic variability and genetic relationships among the major morphological and yield-associated traits paves the first step for any breeding program [31]. The agronomic features and yield attributes are primarily quantitative characters influenced by both genetic and environmental factors. The interactions between genotype and environment have been found in several crops [32,33,34]. Therefore, we have evaluated 34 potential genotypes for 3 locations for variability, principal component, and cluster studies. In general, the phenotypic and genotypic variances were often comparable for all the characters in three locations, illustrating that all the attributes were under governance by genotypic variance. The trait had higher PCV values than corresponding GCV values with very small differences (RD), which implies that genetic factors primarily control these traits rather than environmental factors [35,36]. The results showed that the PCV value was greater than the GCV value for all the studied traits, implying that all the traits were influenced by the environment to some extent among rice genotypes. GCV and PCV values were high for GP, TILL, and PPH compared to the other variables. The closer PCV and GCV values were found in X50F, PL, and PH in three locations. The result reflected that those traits were slightly influenced by environmental factors, indicating that the selection would be rewarding for future breeding programs. This type of result was recorded in rice by other researchers [37,38,39].

The higher value of ECV for the traits TILL, PPH, GP, YPP, and YTH showed that the environment had more impact on the expression of these traits. However, X50F, PH, and Fertility percentage were found to have the very lowest ECV values, indicating lower environmental effects. A similar statement was reported in the findings of [40]. The estimation of heritability with genetic advances would be an effective approach in a selective breeding program [41]. The presence of a high genetic advance value and a high heritability indicates the trait is governed by additive gene action and would be particularly effective in terms of selection and accuracy. The GP, PPH, and TILL have high heritability and GA, suggesting that these variables are controlled by additive genes with little environmental influence. On the other hand, the smaller heritability and GA indicated that traits were influenced by non-additive genes (with more contribution from the environment). For future breeding programs, traits exhibiting high GCV, heritability, and GA should be prioritized [42].

### 4.2. Trait Association

Significant selection promoting genotypes only marked on yield may not be beneficial due to having some environmental impact on polygenic characteristics. As a result, the selection must be made through interrelated traits to improve yield or plant morphology. Thus, correlation analysis was performed among the yield, with the yield contributing ten characters. Yield is assumed to play an essential role in crop production, and several studies have been initiated to examine the association between yield and different yield-related attributes in various crops. In this study, YTH had a positive and significant association (*p* ≤ 0.01 and *p* ≤ 0.001) with GP and YPP, but no significant association with TGW. The results were consistent with the report of [6,43] in rice. There was no correlation between 100-seed weight and yield in Adzuki beans [44], which was in accordance with our results. Debsharma et al. [6] examined 16 germplasms of elite rice and found that PL and TILL are negatively associated with yield, which agreed with these findings. There was a positive correlation between X50F and YTH, indicating that the longer-duration varieties generate higher yield. It has been noted that a positive relationship is observed between PL and PH [45]. The X50F was found to be negatively associated with PH, which corroborated the report of Prasad et al. [46]. It has been reported that YPP exhibited a significantly positive association (*p* ≤ 0.01) with GP, which was also noticed by Debsharma et al. [6]. As a result of their significant impact on yield, GP and YTH should receive priority attention in any rice improvement efforts.

### 4.3. Principal Component Analysis among Traits

Principal Component Analysis (PCA) is a widely used technique to explore the maximum variability through dimensionality reduction from a large number of components [47]. The PCA1, PCA2, PCA3, and PCA4 had eigen values of 27.2%, 18.3%, 14.7%, and 13.3% respectively. Together, they accounted for 73.5% variability of the genotypes used for the diversity analysis. By analysing 31 rice germplasms, Pokhrel et al. [48] found that the first 4 components were responsible for 73.8% of the total variation, which is quite comparable with our findings. PCA-biplots integrate characteristics and objects in two dimensions and reduce conflicting variations, facilitating the identification of the primary characters in datasets [49]. The YTH, YPP, TILL, PPH, and X50F were the top contributors, and the quality of representations elaborates on the total variations because of the highest positive values in the first five PCAs, which is the line in the studies of [50,51]. Those characters might be given keen emphasis when choosing parents.

### 4.4. Heatmap-Oriented Cluster Analysis among Genotypes and Traits

In general, an analysis of clustering is performed to choose the best parents for exploiting high heterotic gain. Based on agro-morphological characteristics, cluster analysis has been previously reported in numerous studies for rice [52,53,54]. In the present study, heatmap-oriented cluster analysis resulted in 3 clusters among 34 elite rice cultivars. The heatmap displays the maximum and minimum values for each genotype for all comparable features in a variety of colours ranging from light hues to deeper intensities. A heatmap scattering analysis showed that cluster II and cluster III had the most genotypes (13), whereas cluster I had the fewest cultivars (8) (Table 6). The rice breeding lines were classified into four clusters, which are depicted in earlier reports by [11,50]. The mean assessment of various clusters for the traits manifested in cluster II genotypes with the PH and PL were accumulated in Group 2, whereas genotypes with TGW, Fertility, TILL, and PPH were more divergent in Group 1. On the other hand, again, cluster II genotypes having GP, YPP, YTH, and X50F were aggregated into Group-3 with high red colour compactness (Figure 7). The cluster-oriented mean values of the mentioned characters were used to estimate the supremacy of the cluster, and therefore, could be assumed in the enhancement of different traits [11,55]. To maximize heterosis and produce a wide variety of segregating populations, Nisar et al. [56] and Ashok et al. [57] advised that genotypes that are used for hybridization be selected from the farthest clusters with good mean accomplishment for studied traits. From cluster analysis, it was recommended that, regarding yield effectiveness and other yield-associated characteristics, elite rice genotypes grouped in clusters II should have more obvious potential for further rice breeding programs.

### 4.5. Multi-Trait Selection Index Based on Factor and MGIDI Index Analysis

Plant breeders typically evaluate a variety of attributes throughout the selection process. A plant ideotype that symbolizes the selection of high-performing plants is kept in mind by plant breeders. By using a stepwise trial-and-error approach, an ideotype gives breeders an ultimate aim for selection, ultimately improving plant performance. The recently introduced MGIDI (multi-trait genotype–ideotypes distance index) is a new approach for selecting genotypes based on multiple trait information [22]. It was simple to identify the genotype’s strengths and weaknesses based on the multiple-trait approach using the MGIDI’s view (Figure 6). The chosen genotypes (Gen2, Gen4, Gen14, Gen22, and Gen30 in Satkhira; Gen2, Gen6, Gen7, Gen15, and Gen30 in Kushtia; and Gen10, Gen12, Gen26, Gen30, and Gen34 in Barishal) had great productivity potential in terms of various yield-contributing traits in addition to grain yield. According to Gabriel et al. [58] and Olivoto and Nardino [22], who evaluated thirteen strawberry genotypes, the Albion cultivar had the highest productivity. In contrast to the selected genotypes of Gen28 and Gen12 in Satkhira, Gen24 and G27 in Kushtia, and Gen24 and Gen21 in Barishal were quite near the cut point (Figure 8), suggesting that this genotype may have advantageous traits. Therefore, when evaluating genotypes, the investigator should pay special attention to those that are quite close to the cut point [22].

## 5. Conclusions

A total of thirty-four elite rice breeding lines were assessed based on ten agronomic and yield-related traits in three locations, revealing that sufficient genetic variation occurred in those studied traits. The majority of the traits under investigation showed significant genetic variability. The GP, TILL, and PPH of all locations exhibited high values of PCV and GCV coupled with GA and heritability. The correlation studies exposed a highly significant positive interrelation between YPP and GP and might be effectively applied as a selection parameter for rice breeding development. The heatmap-oriented cluster analysis exposed a group of 34 genotypes into 3 clusters, displaying significant genetic variability among them. The genotypes accumulated in cluster II performed well in terms of agro-morphological traits. Thus, genotypes Gen2, Gen4, Gen14, Gen22, and Gen30 in Satkhira; Gen2, Gen6, Gen7, Gen15, and Gen30 in Kushtia; and Gen10, Gen12, Gen26, Gen30, and Gen34 in Barishal might be assumed as superior parents based on the MGIDI selection index. Additionally, a heatmap was used to estimate the association matrix between each elite cultivar and its attributes. With the help of the diversity analysis, it will be possible to select the finest recombinants for various traits and to modify these traits in subsequent segregates, which will provide valuable information for further trait-oriented breeding programs or use these selected germplasms as potential breeding material in crossing programs for the development of cultivars suitable for multiple environments under the future changing climate.

## Figures and Tables

**Table 1:** Monthly mean agro-climatic data during the field experiment period in Barishal, Kushtia, and Satkhira.

Locations	Month	Maxi. (°C)	Min. (°C)	Wind (km/h)	Rainfall (mm)	Humidity (%)	Cloud (%)	Pressure (mb)
Barishal	February	27.0	17.7	10.5	0	43.7	0	1014.5
	March	31.7	21.5	9.5	0	54.0	9.7	1013.5
	April	31.5	22.7	11.7	1.0	61.0	13.7	1013.2
	May	35.2	26.7	13.7	2.4	67.7	37.5	1007.7
Kushtia	February	28.0	16.5	10.0	0	39.5	0	1014.7
	March	33.0	20.5	9.5	0	38.0	3.7	1011.7
	April	35.2	21.7	9.2	1.2	42.5	5.2	1009.7
	May	38.5	26.7	12.2	7.0	55.5	33.0	1005.5
Satkhira	February	27.5	17.0	12.2	0	38.5	0	1015.5
	March	33.7	20.7	8.5	0.0	43.7	8.7	1013.2
	April	34.5	22.5	10.2	0.3	50.2	4.7	1010.7
	May	37.2	26.2	15.2	0.1	63.5	36.2	1007.0

**Table 2:** The designation and parentages of 34 elite rice cultivars utilized in the current investigation.

Code	Designation	Parentage
Gen1	BR7671-37-2-2-37-3-P3	BRRI dhan29/IR68144
Gen2	BR8626-19-5-1-2	BR7166-5B-6/SHEW WAR TUN//BRRI dhan47
Gen3	BR8626-10-5-1	BR7166-5B-6/SHEW WAR TUN//BRRI dhan47
Gen4	BR8109-29-2-2-3	BM9821/Parija
Gen5	BR(Bio)8333-BC5-1-1	BRRI dhan29/IRBB60
Gen6	BR(Bio)8333-BC5-1-20	BRRI dhan29/IRBB60
Gen7	BR(Bio)8333-BC5-2-16	BRRI dhan29/IRBB60
Gen8	BR(Bio)8333-BC5-2-22	BRRI dhan29/IRBB60
Gen9	BR(Bio)8333-BC5-3-10	BRRI dhan29/IRBB60
Gen10	BR8631-12-3-5-P2	BR7166-5B-5/BG305//BRRI dhan29
Gen11	BR8631-12-3-6-P3	BR7166-5B-5/BG305//BRRI dhan29
Gen12	BR7831-59-1-1-4-5	BR7166-5B-5/BG305//BRRI dhan29
Gen13	BR8253-9-3-3-1	BR7305-21-6-1/BRRI dhan29//BR7305-21-6-1
Gen14	BR8609-2-B-9-1-B5	BRRIdhan29/BR7166-5B-1
Gen15	BR7815-18-1-3-2-1	BRRI dhan36/BR6725-15-1-2
Gen16	BR7671-37-2-2-3-7-3-P10	BRRI dhan29/IR68144
Gen17	BR7671-37-2-2-3-7-3-P11	BRRI dhan29/IR68144
Gen18	BR8079-19-1-5-1	IR71730-51-2/Nayanmoni
Gen19	BR8590-5-2-5-2-2	BR6902-51-3-2-1/BRRI dhan HR7
Gen20	BR8590-5-3-3-4-2	BR6902-51-3-2-1/BRRI dhan HR7
Gen21	BR8608-39-2-1	BRRI dhan 29/Shaheen Basmati/BR6902-51-3-2-1
Gen22	BRC266-5-1-1-1	BR16/90060-TR1252-8-2-1
Gen23	BRC266-5-1-2-1	BR16/90060-TR1252-8-2-1
Gen24	BR8523-36-2-2-6	IR 77512-128-2-1-2/BR 6817-25-2-2
Gen25	BR8938-19-4-3-1-1	IRBB60/BRRI dhan29
Gen26	BR8333-15-3-2-2	BRRI dhan29/IRBB60
Gen27	IRBB60	Near isogenic line of IR24
Gen28	BRRI dhan28	BR6(IR28)/Purbachi
Gen29	BRRI dhan58	Somaclonal line of BRRI dhan29
Gen30	BRRI dhan29	BG90-2/BR51-46-5
Gen31	BR16	IR1416-131-5/IR1364-37-3-1//IR1544A-E666
Gen32	BRRI dhan50	BR30/IR67684B
Gen33	BRRI dhan63	Amol-3/BRRI dhan28
Gen34	BRRI dhan74	BRRI dhan29/IR68144

**Table 3:** Combined analysis of variance, mean sum of square (MSS) and F-value of the ten traits, and their interaction in elite rice breeding lines in studied locations.

Traits ^a^	Barishal		Kushtia		Satkhira		Pooled ^b^		Interaction ^c^	
	MSS	F-Value	MSS	F-Value	MSS	F-Value	MSS	F-Value	MSS	F-Value
X50F	196.2	160.3 **	66.9	62.7 **	51.7	49.2 **	224.2	203.9 **	44.7	40.7 **
PH	391.3	23.1 **	225.8	21.2 **	258.2	26.8 **	783.4	63.1 **	45.9	3.7 **
TILL	18.7	5.9 **	31.4	4.3 **	10.2	11.2 **	36.3	9.5 **	12	3.2 **
PPH	21.8	7.3 **	8.4	2.6 **	12.3	8.4 **	16.3	6.4 **	13	5.2 **
PL	4.8	5.7 **	8.8	3.7 **	5.4	6.1 **	11.3	8.2 **	3.8	2.8 **
GP	27,549.9	7.5 **	9620.6	11.6 **	7675.9	7.9 **	31,784.3	17.4 **	6531	3.6 **
Ferti	258.1	14.1 **	161.6	6.4 **	124.7	5.4 **	171.3	7.7 **	186.8	8.4 **
YPP	27.2	12.2 **	43.7	7.9 **	16.2	10.4 **	48.9	15.8 **	19	6.2 **
TGW	37.2	19.2 **	27.3	36.8 **	20.7	6.3 **	72.7	36.5 **	6.2	3.2 **
YTH	1.9	10.5 **	2.9	9.3 **	1.8	16.8 **	4.8	22.6 **	1	5.2 **

** Significant at a probability level of 1%, ^a^ abbreviation of traits is as per given under material and method, ^b^ data of three locations pooled and analysed; ^c^ genotype × Location interaction, MSS = Mean sum of square. Genotypes df is 33 and rep df is 2. Notes: X50F = days to 50% flowering, PH = plant height (cm), TILL = number of tillers per hill, PPH = number of panicles per hill, PL = panicle length (cm), GP = grains per panicle, Ferti = fertility (%), YPP = grain yield per plant (gm), TGW = thousand grain weight (gm), and YTH = grain yield (t/ha).

**Table 4:** Estimation of variance components, broad sense heritability, and genetic advance in elite rice genotypes.

Traits	Location	X50F	PH	TILL	PPH	PL	GP	Ferti	YPP	TGW	YTH
Range	Barishal	103–135	80–148	8–23	8–23	20–27.9	151–607	51–87.3	16.9–32.5	15.4–33	4.5–8.6
	Kushtia	110–129	77–131	8–29	5–18	19–30	160–484	57.4–94.9	18–37.5	16.9–31.2	4.4–9.3
	Satkhira	106–124	81–124	7–18	6.3–16.5	18.5–29.8	79–375	54.3–94.5	17.6–28.9	13.8–28.9	4.6–7.9
Mean ± SE	Barishal	118.2 ± 0.9	105.7 ± 3.4	13.8 ± 1.5	12.5 ± 1.4	23.9 ± 0.7	307.3 ± 49.6	66.1 ± 3.4	24.2 ± 1.2	21.8 ± 1.2	6.41 ± 0.4
	Kushtia	121.0 ± 0.8	99.0 ± 2.67	15.7 ± 2.2	10.4 ± 1.5	24.4 ± 1.3	267.5 ± 23.5	79.7 ± 4.2	28.6 ± 1.9	22.1 ± 0.7	7.04 ± 0.5
	Satkhira	114.7 ± 0.8	101.2 ± 2.5	11.2 ± 0.7	10.7 ± 0.9	23.8 ± 0.7	210 ± 25.5	80.2 ± 3.9	22.7 ± 1.1	20.7 ± 1.5	6.24 ± 0.2
ẟ^2^g	Barishal	64.9	124.7	5.2	6.3	1.3	7951.9	88.9	8.30	11.7	0.6
	Kushtia	21.9	71.7	8.1	1.7	2.2	2929.7	45.4	12.7	8.8	0.8
	Satkhira	16.8	82.8	3.1	3.6	1.5	2234.5	33.9	4.8	5.8	0.5
ẟ^2^p	Barishal	65.4	130.4	6.2	7.3	1.6	9183.3	95.0	9.1	12.4	0.6
	Kushtia	22.3	75.3	10.5	2.8	2.9	3206.8	53.8	14.6	9.1	0.9
	Satkhira	17.3	89.0	3.4	4.1	1.8	2558.6	41.5	5.4	6.9	0.6
ẟ^2^e	Barishal	1.3	16.9	3.2	3.0	0.8	3694.2	18.3	2.3	1.9	0.2
	Kushtia	1.1	10.7	7.4	3.2	2.4	831.5	25.5	5.5	0.7	0.3
	Satkhira	1.1	9.6	0.9	1.5	0.8	972.4	22.9	1.5	3.3	0.1
GCV (%)	Barishal	54.9	117.9	37.3	50.3	5.6	2588.0	134.5	34.4	53.6	9.3
	Kushtia	3.8	8.5	17.9	12.7	5.9	20.3	8.5	12.5	13.4	13.2
	Satkhira	3.5	8.9	15.7	17.6	5.2	22.5	7.3	9.7	11.6	11.9
PCV (%)	Barishal	6.8	10.5	16.5	20.0	4.8	29.0	13.5	11.9	15.6	12.0
	Kushtia	3.96	9.2	24.8	8.7	22.9	10.5	14.9	13.9	15.4	8.7
	Satkhira	3.6	9.5	17.8	6.4	26.9	9.4	11.1	14.5	13.0	6.4
ECV (%)	Barishal	6.8	11.3	20.9	6.1	35.1	14.9	13.4	16.9	13.7	6.1
	Kushtia	0.8	3.3	17.2	17.1	6.3	10.7	6.3	8.1	3.8	7.9
	Satkhira	0.8	3.0	8.5	11.2	3.9	14.8	5.9	5.4	8.7	5.2
RD (%)	Barishal	0.9	3.9	13.0	13.8	3.8	19.7	6.4	6.1	6.3	6.7
	Kushtia	1.6	4.7	23.3	37.5	27.2	8.6	15.7	12.5	2.7	10.7
	Satkhira	2.2	6.9	8.8	11.9	16.4	12.7	18.4	9.5	16.7	5.9
h^2^b (%)	Barishal	99.4	95.6	83.2	86.2	82.6	86.6	93.5	91.7	94.8	90.5
	Kushtia	98.4	95.3	76.7	62.5	72.8	91.4	84.2	87.4	97.3	89.2
	Satkhira	97.9	93.0	91.2	88.0	83.6	87.3	81.6	90.5	84.1	94.0
GA (%)	Barishal	14.0	21.3	30.7	38.4	9.1	55.6	28.4	23.5	31.4	23.5
	Kushtia	7.9	17.2	32.5	20.7	10.5	39.8	15.9	24.1	27.3	25.7
	Satkhira	7.3	17.8	30.9	34.0	9.7	43.3	13.5	19.0	21.9	23.9

X50F = days to 50% flowering, PH = plant height (cm), TILL = number of tillers per hill, PPH = number of panicles per hill, PL = panicle length (cm), GP = grains per panicle, Ferti = fertility (%), YPP = grain yield per plant (gm), TGW = thousand grain weight (gm), and YTH = grain yield (t/ha).

**Table 5:** List of the best number of clusters and their index value of 30 indices implemented in the NbClust package in R.

	KL	CH	Hartigan	CCC	Scott	Marriot
No. of clusters	8	3	3	10	3	3
Index value	2.1197	7.8206	3.051	−0.7325	87.9885	2.49 × 10^12^
	TrCovW	TraceW	Friedman	Rubin	Cindex	DB
No. of clusters	3	3	8	8	8	10
Index value	310.342	23.6767	61.487	−0.1678	0.4	1.0447
	Silhouette	Duda	PseudoT2	Beale	Ratkowsky	Ball
No. of clusters	10	2	2	2	4	3
Index value	0.2796	1.0678	−1.6513	−0.4058	0.3152	61.7704
	PtBiserial	Gap	McClain	γ	Gplus	Tau
No. of clusters	5	2	2	10	10	5
Index value	0.5448	−0.7636	0.8023	0.8943	2.8324	79.1515
	Frey	Hubert	Dindex	Dunn	SDindex	SDbw
No. of clusters	6	9	3	10	7	10
Index value	0.6385	0.0111	2.4227	0.5084	0.8697	0.3125

**Table 6:** Clustering of 34 elite rice breeding lines.

Cluster	Genotypes Number	Name of Genotypes Code
I	8	Gen1, Gen2, Gen3, Gen4, Gen8, Gen9, Gen23, Gen29
II	13	Gen6, Gen7, Gen12, Gen15, Gen18, Gen19, Gen24, Gen28, Gen30, Gen31, Gen32, Gen33 Gen34
III	13	Gen5, Gen10, Gen11, Gen13, Gen14, Gen16, Gen17, Gen20, Gen21, Gen22, Gen25, Gen26, Gen27

**Table 7:** Original value (Xo), selected value (Xs), selection differential in percentage (SDperc), heritability (h^2^), and selection gain in percent (SGperc) for the MGIDI in 34 rice genotypes in Satkhira.

Variable	Factor	Xo	Xs	SDperc	h^2^	SGperc	Sense	Goal
PPH	FA1	10.7	11.8	9.88	0.88	8.7	increase	100
GP	FA1	210	244	16.3	0.873	14.3	increase	100
TGW	FA1	20.7	20.4	−1.88	0.841	−1.58	increase	0
X50F	FA2	115	115	−0.117	0.98	−0.115	increase	0
PH	FA2	101	101	−0.276	0.963	−0.266	increase	0
TILL	FA2	11.2	11.5	2.82	0.91	2.57	increase	100
YPP	FA3	22.8	25.6	12.4	0.904	11.2	increase	100
YTH	FA3	6.24	6.67	6.84	0.941	6.43	increase	100
PL	FA4	23.8	25.1	5.29	0.836	4.42	increase	100
Fertility	FA4	80.2	76.9	−4.12	0.816	−3.36	increase	0

**Table 8:** Original value (Xo), selected value (Xs), selection differential in percentage (SDperc), heritability (h^2^), and selection gain in percent (SGperc) for the MGIDI in 34 rice genotypes in Kushtia.

Variable	Factor	Xo	Xs	SDperc	h^2^	SGperc	Sense	Goal
YPP	FA1	28.6	31.9	11.4	0.874	9.96	increase	100
YTH	FA1	7.04	7.88	11.9	0.892	10.6	increase	100
TILL	FA2	15.7	16	1.89	0.767	1.45	increase	100
PPH	FA2	10.4	10.4	0.0471	0.624	0.0294	increase	100
GP	FA2	267	267	−0.108	0.914	−0.0989	increase	0
X50F	FA3	121	124	2.31	0.984	2.28	increase	100
PH	FA3	99.1	105	6.01	0.952	5.72	increase	100
TGW	FA4	22.2	24.1	8.6	0.973	8.36	increase	100
PL	FA5	24.4	25.2	3.48	0.728	2.53	increase	100
Fertility	FA5	79.8	78.1	−2.03	0.842	−1.71	increase	0

**Table 9:** Original value (Xo), selected value (Xs), selection differential in percentage (SDperc), heritability (h^2^), and selection gain in percent (SGperc) for the MGIDI in 34 rice genotypes in Barishal.

Variable	Factor	Xo	Xs	SDperc	h^2^	SGperc	Sense	Goal
YPP	FA1	24.2	26	7.75	0.918	7.11	increase	100
YTH	FA1	6.41	6.93	8.20	0.905	7.42	increase	100
TILL	FA2	13.9	15	8.31	0.827	6.87	increase	100
PPH	FA2	12.5	14.1	12.6	0.863	10.8	increase	100
PH	FA3	106	112	5.92	0.956	5.66	increase	100
GP	FA3	307	323	5.07	0.866	4.39	increase	100
PL	FA3	23.9	24.9	4.15	0.826	3.43	increase	100
Fertility	FA3	66.1	60.1	−9.06	0.929	−8.41	increase	0
X50F	FA4	118	114	−3.79	0.994	−3.77	increase	0
TGW	FA4	21.9	23.4	6.96	0.948	6.6	increase	100

## Data Availability

Not applicable.
